# Editorial: Global excellence in nuclear medicine: North America

**DOI:** 10.3389/fmed.2023.1300179

**Published:** 2023-10-25

**Authors:** Abhishek Jha, Ali Cahid Civelek

**Affiliations:** ^1^Section on Medical Neuroendocrinology, *Eunice Kennedy Shriver* National Institute of Child Health and Human Development, National Institutes of Health, Bethesda, MD, United States; ^2^Nuclear Medicine, Radiology, and Radiological Science, Johns Hopkins Medicine, Baltimore, MD, United States

**Keywords:** collaboration, flutriciclamide, [18F]GE-180 PET, PET, PET/CT, PET/MRI, quantitative susceptibility mapping (QSM), nuclear medical imaging

In this editorial, the significance of global cooperation in promoting scientific knowledge, particularly in the field of Nuclear Medicine, is discussed. Frontiers in Medicine has arranged a special edition that features the work of distinguished researchers from North America and showcases their recent innovations in Nuclear Medicine.

Effective communication of findings is a critical aspect of scientific progress, especially when it comes to patient care. The imaging report plays a vital role in this communication process. In their review, Murad et al. highlighted the need for standardized classification schemes and reporting guidelines for PET/CT scans using ^18^F-FDG, ^68^Ga-DOTA-peptides, and PSMA-based radiopharmaceuticals in oncology. These schemes ensure that imaging reports are clear, concise, and evidence-based, which makes clinical decision-making easier. The paper discusses various classification schemes for interpreting and reporting PET imaging for lymphoma, multiple myeloma, melanoma, head and neck cancers, prostate cancer, and neuroendocrine tumors. The review also mentions the novel PET Response Evaluation Criteria for Immunotherapy (PERCIMT). However, the authors caution that the implementation of these recently proposed interpretation schemes to new indications would require validation in further prospective studies before they can be integrated into routine clinical practice.

Two articles in this series analyze the issue of diagnostic radiopharmaceutical extravasations during nuclear medicine procedures (Osborne et al.; Crowley et al.). The review article by Osborne et al. challenges the long-standing assumptions regarding diagnostic radiopharmaceutical extravasations. According to the article, significant extravasations can harm patients and irradiate tissue beyond acceptable limits. The findings are based on a review of 58 peer-reviewed documents that suggest patient harm due to extravasation and evaluation of five extravasated patients who underwent repeat imaging at the authors' institutions. The study challenges the belief that diagnostic extravasations do not cause harm and emphasizes the need for monitoring and dosimetry of extravasated tissue in some cases. The study also recommends process improvements to reduce the frequency of extravasations in nuclear medicine procedures. This is important because quantification is essential in precision medicine, and we need to follow ALARA principles. The case report by Crowley et al. highlights the importance of detecting the excess presence of the radiopharmaceutical 99mTc-MDP near the injection site during nuclear medicine procedures. Detecting extravasations or venous stasis early in the injection process is crucial for ensuring good quality of images and minimizing radiation dose to surrounding tissue. This case emphasizes the need for proactive measures to mitigate potential issues during radiopharmaceutical administration. Early detection of the excess presence of 99mTc-MDP near the injection site enables technologists to apply mitigation tactics early in the process. Detecting extravasation or stasis early in the injection process can be necessary for image interpretation and minimizing radiation dose to tissue.

Kim et al. conducted a study to evaluate cerebrovascular malformations with the help of PET/MRI scan and a radiotracer flutriciclamide ([18F]GE-180). The study aimed to examine the role of neuroinflammation in cerebral cavernous malformations (CCM), investigate the uptake of flutriciclamide in different brain regions in CCM patients (*n* = 5) and controls (*n* = 6), and compare the uptake with iron deposition assessed by quantitative susceptibility mapping (QSM). The study concluded that the uptake of flutriciclamide in CCM lesions is positively correlated with QSM. Therefore, flutriciclamide can be used as a potential marker for neuroinflammation in CCM patients. Flutriciclamide uptake combined with QSM offers complementary information for lesion delineation. It highlights the benefits of hybrid modalities like PET/MRI that can not only locate CCM lesions but also quantify both neuroinflammation and iron deposition simultaneously.

Collaboration on a global scale is critical for advancing scientific knowledge, particularly in the field of nuclear medicine. International researchers play a vital role in contributing to this field. Effective communication in patient care relies on standardized reporting guidelines. The literature continues to feature publications that demonstrate these ongoing efforts (1–3). Detecting and addressing extravasations during procedures is essential to prevent harm to patients and exceeding radiation limits. Radiopharmaceutical research, such as flutriciclamide, shows promise for a better understanding of diseases like CCM, and the optimization of simultaneous PET/MRI in performing multiparametric evaluation in oncology, specifically in brain, head and neck, breast, liver, colorectal, gynecologic, prostate, and bladder tumors, particularly in the pediatric population (4–24). Leading societies in nuclear medicine (SNMMI and EANM) and MRI (ISMRM) have released a consensus recommendation for PET/MRI in oncology (25), as well as recently, SNMMI, ACNM, and ASNC released a joint position statement on extravasations of diagnostic radiopharmaceuticals and medical event reporting (26). They summarized that the extravasations are best managed on an institutional level and do not require any additional NRC regulation. These extravasations do not cause issues in patient-safety but rather a quality-control issue as they may affect quality of diagnostic images, especially for quantitative studies. These indicate ongoing efforts to enhance the quality and safety of Nuclear Medicine practices through research and collaboration.

## Author contributions

AJ: Writing—original draft, Writing—review and editing. AC: Writing—review and editing.

## Author disclaimer

This work was done as part of the AJ's official duties as an NIH employee and is a Work of the United States Government.
